# An Intrinsic Optical Fiber Uric Acid Sensor Using a Molecularly Imprinted Chitosan Membrane-Coated Optical Fiber as a Transducer

**DOI:** 10.3390/s26196239

**Published:** 2026-10-01

**Authors:** Geraldine Medina, Shiquan Tao

**Affiliations:** Department of Chemistry and Physics, West Texas A&M University, WTAMU Box 60732, Canyon, TX 79106, USA

**Keywords:** optical fiber chemical sensor, molecularly imprinted chitosan, uric acid, solid phase microextraction, UV/Vis absorption spectrometry, bent optical fiber probe

## Abstract

**Highlights:**

- Solid phase microextraction integrated with fiber optic spectroscopy
- Molecularly imprinted chitosan membrane as SPME material
- MIC-SPME increases fiber optic uric acid sensor’s sensitivity by 1.0 × 10^4^ time
- MIC-SPME blocks out interference species and improving sensor’s selectivity
- Selective response to uric acid, but insensitive to urate anions

**Abstract:**

An intrinsic optical fiber uric acid (UA) sensor has been developed using a molecularly imprinted chitosan (MIC) membrane-coated optical fiber as a transducer. The MIC was prepared by crosslinking chitosan with glutaraldehyde as a crosslinking reagent and UA as the template molecule. After an MIC membrane was coated on the surface of a bent optical fiber probe (BOFP), the template UA molecules were washed out of the membrane by using a diluted ammonia solution (0.10 M). This MIC membrane-coated BOFP was deployed into a sample solution for analyzing UA concentration. UA in the sample solution was selectively extracted/concentrated into the MIC membrane, while interference species in the sample solution were blocked from entering into the membrane. UA extracted into the MIC membrane was detected by passing a light beam through the BOFP and the intrinsic optical absorption signal at 315 nm by UA itself was detected as a sensing signal. There is no chemical reaction involved, and no chemical reagent is needed when using this sensor to analyze UA in a sample solution. Compared with a bare fiber BOFP, MIC membrane extraction of UA from a sample solution increased the sensor’s sensitivity by 1.0 × 10^4^ times. The sensor achieved a detection limit of 0.049 µg/mL with a linear calibration concentration that ranged up to 5.0 µg/mL. The sensor demonstrates a good precision in analyzing UA in a urine sample with RSD% = 2.9%. This is a selective sensor; species potentially co-existing in regular urine samples with UA were tested, and were found not to cause interference. This sensor selectively responds to UA in its acid form, but is not sensitive to urate anions, which is different from most reported methods for analyzing UA. The sensor has been tested for analyzing UA in a urine sample and the obtained results demonstrate the feasibility of this sensor in real-world applications of analyzing UA in urine samples.

## 1. Introduction

Uric acid (UA) is a significant component in urine and blood serum. The concentration of this compound in urine and blood serum samples is an indicator of potential diseases, including kidney stones, gout, cardiovascular stress and metabolic disorder [1,2,3]. In present clinical labs, UA concentration in urine and blood serum samples is analyzed by using an enzymatic colorimetric method [4]. Uricase selectively catalyzes UA oxidation, and hydrogen peroxide is a product of that catalyzed oxidation reaction. The generated hydrogen peroxide was catalyzed by another enzyme, peroxidase, to react with 4-aminoantipyrine and generate a red-colored quinoneimine dye which is analyzed with UV/Vis absorption spectrometry to determine UA concentration in the original sample solution. This method involves two enzymes which are expensive, and takes about 30 min to get analytical results at room temperature operation.

UA concentration in urine and blood serum samples ranges from a few mg/L to hundreds of mg/L, depending on several factors, including age, health condition, diet, and how much water was drunk [2,3]. Therefore, UA concentration in a randomly collected urine sample was not used in medical diagnosis as an appropriate disease indicator [3,5]. It is a normal medical practice to collect all released urine within 24 h from a patient and analyze total UA quantity in the urine samples collected during the 24 h [5]. Some additional sample treatments are required in order to use this traditional method for analyzing UA, including: (1) the newly collected urine sample has to be pre-treated to quench bioactivity in the urine sample; (2) NaOH has to be added to adjust the pH value of the newly collected urine sample solution in order to prevent UA from precipitating out; and (3) the treated sample has to be stored in a refrigerator to keep sample temperature at 4–6 °C before analysis [3,5]. For a hospitalized patient, a nurse can conduct the sample treatments and store the sample before analysis. However, it is difficult for this practice to be applied to those patients who are not hospitalized. Therefore, a technique which can be used for conveniently analyzing a urine sample’s UA concentration outside of traditional clinical labs is very attractive.

In recent years, there have been some efforts to develop a point-of-care (POC) device for analyzing UA in urine samples outside of traditional clinical labs [6,7]. For example, electrochemical sensors using disposable low-cost electrodes have been investigated for use in analyzing UA in urine or saliva samples [8]. UA can be oxidized on different electrodes to generate current signal, which can be used to quantitatively analyze this compound [8,9,10,11,12]. A common problem with electrochemical methods for analyzing UA in a urine sample is interference from co-existing species in urine. Although intensive efforts have been made to modify electrodes with different compounds, metal nanoparticles, and carbon nanotubes, the outcome of these efforts was limited [8,9,10,11,12]. Optical spectroscopic techniques, such as UV/Vis absorption spectrometry, fluorescence spectrometry, surface plasmonic spectrometry, and chemiluminescence have also been investigated as a means of developing sensors to analyze UA for POC applications [13,14,15,16,17,18,19]. Some of these techniques are very sensitive and can achieve a detection limit at the µM-to-sub-µM level [13,14], which is more-than-needed sensitivity for analyzing UA in urine samples. However, spectral interference by co-existing species in urine samples presents a challenge in applying these techniques to POC applications. There have also been efforts to impregnate papers with the enzymes and reagents used in the traditional clinical enzymatic test, and to use these reagent-impregnated papers as reactors to analyze UA in urine samples for POC applications [19,20,21]. In such cases, a mobile device, such as a cellphone, can be used as a detector to detect the color intensity of the formed reaction product. Although these paper-based detectors are easy to use, the precision of these detectors is a concern.

In addition to medical diagnosis, UA analysis has other applications. In environmental monitoring, elevated UA concentration in river and estuary water samples is a reliable indicator of domestic sewage pollution or agricultural run-off, because unpolluted natural water has very limited UA [22,23]. High UA concentration in food products is an indicator of food spoilage or insect contamination [24]. In addition to traditional enzymatic methods, electrochemical detection, mass spectrometry, UV/Vis spectrometry, and fluorescence spectrometry have been used to detect UA concentration in environmental and food samples [25,26]. However, a separation procedure, such as HPLC [27,28], capillary electrophoresis [29] or solid phase microextraction (SPME) [30,31], has to be employed to isolate UA from the complex sample matrix in order to use these methods to analyze UA concentration in environmental and food samples.

**Figure 1:**
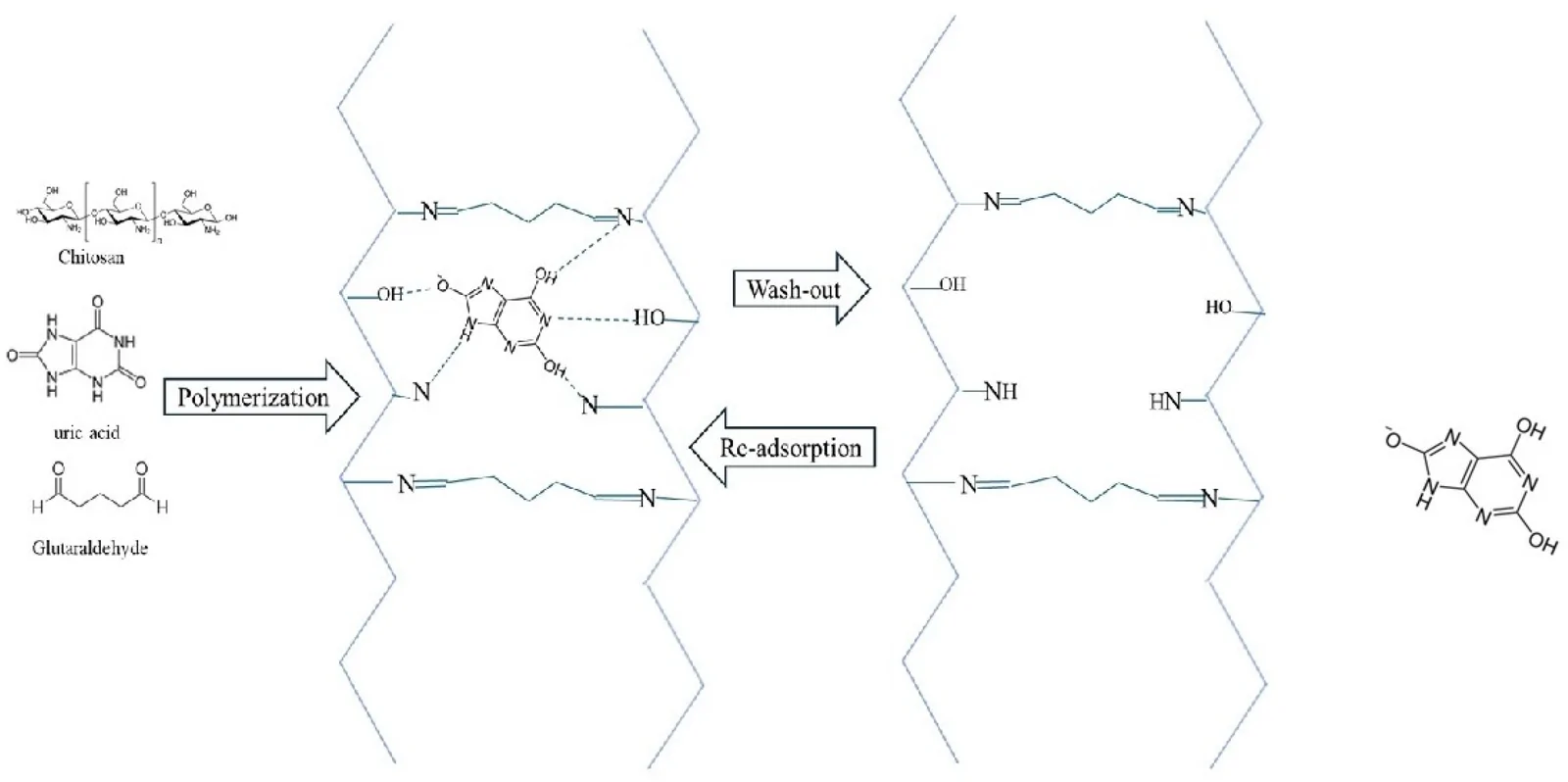
A diagram illustrating the formation of MIC from chitosan, GTA and UA, washing out of UA from formed MIC, and selective re-adsorption of UA into MIC.

In our recent works, we integrated a selective SPME separation technique with optical spectrometry into a single optical fiber to develop highly sensitive and selective optical fiber chemical sensors (OFCSs) for analyzing trace weak organic acids in samples of complex sample matrices [32,33,34,35,36]. Molecularly imprinted chitosan (MIC) membranes have been used as SPME materials for selectively extracting/concentrating target analytes from sample solutions, while blocking out interference species from entering the membrane. Chitosan is a polysaccharide hydrogel from a natural resource (shrimp shell). This polymer can absorb a large quantity of water due to the existence of hydrophilic groups (-NH_2_, -OH) as its side function groups. Weak organic acids can react with the -NH_2_ group in this polymer and be extracted/concentrated into the polymer. When chitosan’s polymer chains are crosslinked with a crosslinking reagent in the existence of a weak organic acid, the weak organic acid is immobilized inside the crosslinked polymer as a template. The immobilized weak organic acid can be washed out of the crosslinked polymer and left behind vacancies inside the polymer. These vacancies have bonding sites specific to the template molecule, and the pore’s geometry is complementary to the template molecule. This makes the MIC highly selective in extracting template weak organic acid, but does not concentrating other compounds, including the template organic acid in its anion form, into the imprinted polymer. Figure 1 diagrammatically shows the formation of a UA-templated MIC from chitosan, glutaraldehyde (crosslinking agent) and UA, and the washing out/selective re-adsorption of UA into MIC as an example.

When such an MIC membrane is coated on the surface of an optical fiber core, the MIC membrane functions as a cladding of the optical fiber to guide light through the fiber. The template weak organic acid extracted from a sample solution into the MIC membrane absorbs the evanescent wave (EW) of light guided through the optical fiber at the weak organic acid’s intrinsic peak absorption wavelength. This intrinsic EW absorption signal can be detected by using an optical fiber-compatible spectrometer as a sensing signal for analyzing the concentration of the weak organic acid in a sample solution. By integrating an MIC membrane as a selective SPME element in this sensing technique, highly selective and sensitive OFCSs have been developed in our recent works for analyzing weak organic acids in samples of complex matrices, such as red wine [32], grape juice, plum juice [33], and green coffee extract [34].

This paper presents our recent effort on the development of a UA sensor using our MIC-EW-OFCS technique. UA was used as a template in synthesizing the MIC membrane on the surface of a bent optical fiber probe (BOFP). After washing out UA from the MIC with a 0.10 M NH_3_ solution, the MIC membrane-coated BOFP was connected to an optical fiber-compatible light source and an optical fiber-compatible spectrometer to form a sensor. The response of this sensor to UA and other chemical species potentially co-existing with UA in urine samples was investigated. The sensor was tested for its ability to analyze UA in a urine sample and the obtained results demonstrate the feasibility of this sensor for analyzing UA in urine samples.

## 2. Materials and Methods

### 2.1. Chemicals

The following chemical reagents used in this work were purchased from Sigma-Aldrich (St. Louis, MO, USA): uric acid (2,6,8-trihydroxypurine, >99%), chitosan (chitosan from shrimp shells, practical grade), glutaraldehyde (GTA, OHC(CH_2_)_3_CHO, 50% aqueous solution), acetic acid (glacial, 99–100%), bovine serum albumin (BSA, lyophilized powder, >98.5%), urea (ACS reagent grade, 99.0–100.5%), D-fructose (C_6_H_12_O_6_, ≥99%), caffeine (anhydrous, 99%), creatinine (anhydrous, ≥98%), D.L-tryptophan (≥98%, HPLC), L-ascorbic acid (99%), ammonium chloride (ACS reagent, ≥99.5%), and potassium chloride (KCl, ≥99.0%). All other reagents used in this work were of analytical reagent grade. Deionized (DI) water was used to prepare all the aqueous solutions used in this work.

A 2% acetic acid solution was prepared by diluting the glacial acetic acid with DI water. A 10 mg/mL chitosan solution in 2% acetic acid was prepared by mixing 0.50 g chitosan with 50 mL of the 2% acetic acid solution with continuous magnetic stirring for >12 h. The original 50% GTA solution was 1:1 diluted with DI water to make a 25% GTA solution. A 1.0 mg/mL uric acid (sodium salt form) solution was prepared by mixing 30 mg uric acid with 20 mL DI water in a small reagent bottle, and drop-by-drop adding 1 M NaOH while shaking the reagent bottle until the solid uric acid powder was completely dissolved. DI water was then added to make the total volume of the solution 30.0 mL.

A UA-templated MIC coating solution was prepared by mixing 1.0 mL of the 10 mg/mL chitosan solution, 0.50 mL of the 1.0 mg/mL UA (sodium salt form) solution and 50 µL of the 25% GTA solution in a small reagent bottle. The mixed solution was vigorously shaken for 1 min before coting the solution on the BOFP surface. In this coating solution, UA should exist in an acidic form although its sodium salt form was added into the mixture solution, because of the existence of excess acetic acid (150 times more than UA sodium salt by number of moles) in the mixed solution. This coating solution was prepared immediately before coating the solution on a BOFP.

A 50.0 µg/mL UA stock standard solution was prepared in two steps due to the limited solubility (60 µg/mL) of uric acid in DI water at room temperature [37]. First, 30.0 mg of reagent UA powder was mixed with 30.0 mL DI water in a reagent bottle and the mixture was vigorously shaken for 3 min. The UA did not completely dissolve because its concentration (1.00 mg/mL) was above this compound’s solubility limit, but a homogenous mixture was obtained. We immediately transferred 1.00 mL of the mixture to another small reagent bottle, added 19.00 mL DI water and shook the reagent bottle to make a 50.0 µg/mL UA stock standard solution, which is a true solution because UA concentration in this solution is below UA’s solubility limit. UA standard solutions of lower concentration used in this work were prepared by diluting this stock standard solution with DI water appropriately.

### 2.2. Instruments

The following instruments were used in this work: an optical fiber-compatible UV/Vis spectrometer (USB4000, OceanOptics, Olando, FL, USA), optical fiber-compatible comb light source (DH-2000), and a Nicolet iS5 FTIR spectrometer (Thermo Fisher Scientific, Madison, WI, USA) with a diamond crystal attenuated total reflection (ATR) accessory. A computer program ((OceanView) from OceanOptics was used to record the fiber optic EW spectrum (including the light intensity spectrum and EW absorption spectrum) as well as to monitor the change in light intensity or fiber optic EW absorbance with time at the selected wavelength(s).

### 2.3. Preparing Membranes of GTA-Crosslinked Chitosan and UA-Templated MIC for FTIR Analysis of the Membranes

Membranes of GTA-crosslinked chitosan and UA-templated MIC were prepared, so that we could identify the immobilization of UA in MIC by using FTIR spectrometry. A GTA-crosslinked chitosan membrane was prepared by mixing 1.9 mL of the 10 mg/mL chitosan solution and 100 µL of the 25% GTA solution. The solution was placed on a glass slide after thorough mixing. The mixture solution was air dried in an ambient air environment for >48 h. A brownish colored film formed on the glass slide surface. A similar method was used to prepare a UA-templated MIC membrane, except that the mixture solution was made by mixing 1.5 mL of the 10 mg/mL chitosan solution, 0.50 mL of the 1.0 mg/mL UA (sodium form) solution and 100 µL of the 25% GTA solution. The formed membranes were removed from the glass slide surface with a sharp blade for FTIR study. Similarly, UA should exist in an acidic form in the MIC membrane, although its sodium salt form was added into the mixture solution due to the existence of acetic acid in the chitosan solution used.

### 2.4. Preparing a BOFP and Coating the BOFP with UA-Templated MIC

A BOFP was used in this work for fiber optic EW absorption spectrometric analysis of UA extracted into the MIC membrane, because fiber optic EW absorption spectrometry has higher sensitivity with a bent optical fiber structure [38,39]. A procedure reported in our previous publications was adopted to prepare the BOFP [32,33,34,35,36]. This procedure involves hand-forcing to bend a straight fiber (FG200AEA, fiber core/cladding diameter = 200/220 µm, Thorlabs, Newtown, NJ, USA) inserted into a small butane flame. The bent part of the BOFP was then washed by using a K_2_CrO_4_/H_2_SO_4_ washing solution (caution: K_2_CrO_4_/H_2_SO_4_ washing solution is a corrosive solution and must be used carefully and waste solution must be disposed following EPA regulations) to eliminate any organic species on its surface. The bent part was then etched to reduce the fiber core diameter in order to increase the sensitivity of fiber optic EW absorption spectrometry [38,39] by inserting the bent probe into a concentrated hydrofluoric acid (HF) solution (caution: concentrated HF solution is poisonous and must be used with caution) for 20 min. The bent part of the HF-etched BOFP was then inserted into a 1 M NaOH solution for 20 min to activate the fiber surface’s -OH group for better coating adhesion, and rinsed with DI water before coating an MIC membrane.

When coating an MIC membrane on the BOFP’s bent part, the probe was simply inserted into the freshly prepared MIC coating solution and pulled out slowly. In the case of repeated coating operations, there was a 30 s waiting time between consecutive coating operations to allow for vaporization of the solvent (DI water). Four dip-coatings were performed while preparing the sensor probe used in this work. The coated probe was dried/gelated at room temperature in an ambient air environment for >12 h before use.

### 2.5. Monitoring Washing out of UA from MIC Membrane with Fiber Optic EW Absorption Spectrometry

The two ends of the MIC-coated BOFP were connected to the USB4000 spectrometer and the DH-2000 light source, respectively, by using subminiature version A connectors. The light intensity spectrum guided through the BOFP was monitored with the OceanView program. When the coated probe was just inserted into DI water in a small reagent bottle, a reference light intensity spectrum was recorded and was used as reference intensity for monitoring the fiber optic EW absorption spectrum. The probe was then placed into a 0.10 M NH_3_ solution and fiber optic EW absorption spectra were recorded at different times.

### 2.6. Testing MIC Membrane-Coated BOFP for Analyzing UA in Aqueous Solution Samples

The bent probe was moved from the 0.10 NH_3_ solution into DI water in a small reagent bottle. After the monitored light intensity spectrum was stabilized, a new reference light intensity spectrum was recorded and was used as reference for fiber optic EW absorption spectrum measurement. The bent probe was then placed into a sample solution, and a fiber optic EW absorption spectrum was recorded 1 min after the probe was inserted into the sample solution. The probe was re-inserted into the 0.10 NH_3_ solution for 4 min to wash out UA adsorbed into the MIC membrane, and then back to DI water to re-generate the probe for the next measurement. The sample solutions tested in this work were at ambient room temperature.

### 2.7. Analyzing UA in a Urine Sample and a Standard Addition Urine Sample

UA concentration in a urine sample donated by a volunteer donor was analyzed with the sensor developed in this work. 19.50 mL DI water was added to 0.500 mL of a freshly collected urine sample to make a test sample. The bent fiber probe was inserted into the test sample solution and a fiber optic EW absorption spectrum was recorded after 1 min.

A standard addition urine sample was prepared by mixing 0.500 mL of the freshly collected urine sample, 2.00 mL of a 10.0 µg/mL UA standard solution and 17.50 mL of DI water. The standard addition sample was then analyzed by inserting the bent fiber probe into the sample solution and recording a fiber optic EW absorption spectrum after 1 min.

## 3. Results and Discussion

### 3.1. FTIR Spectra Confirming UA Imprinting in the MIC Membrane

**Figure 2:**
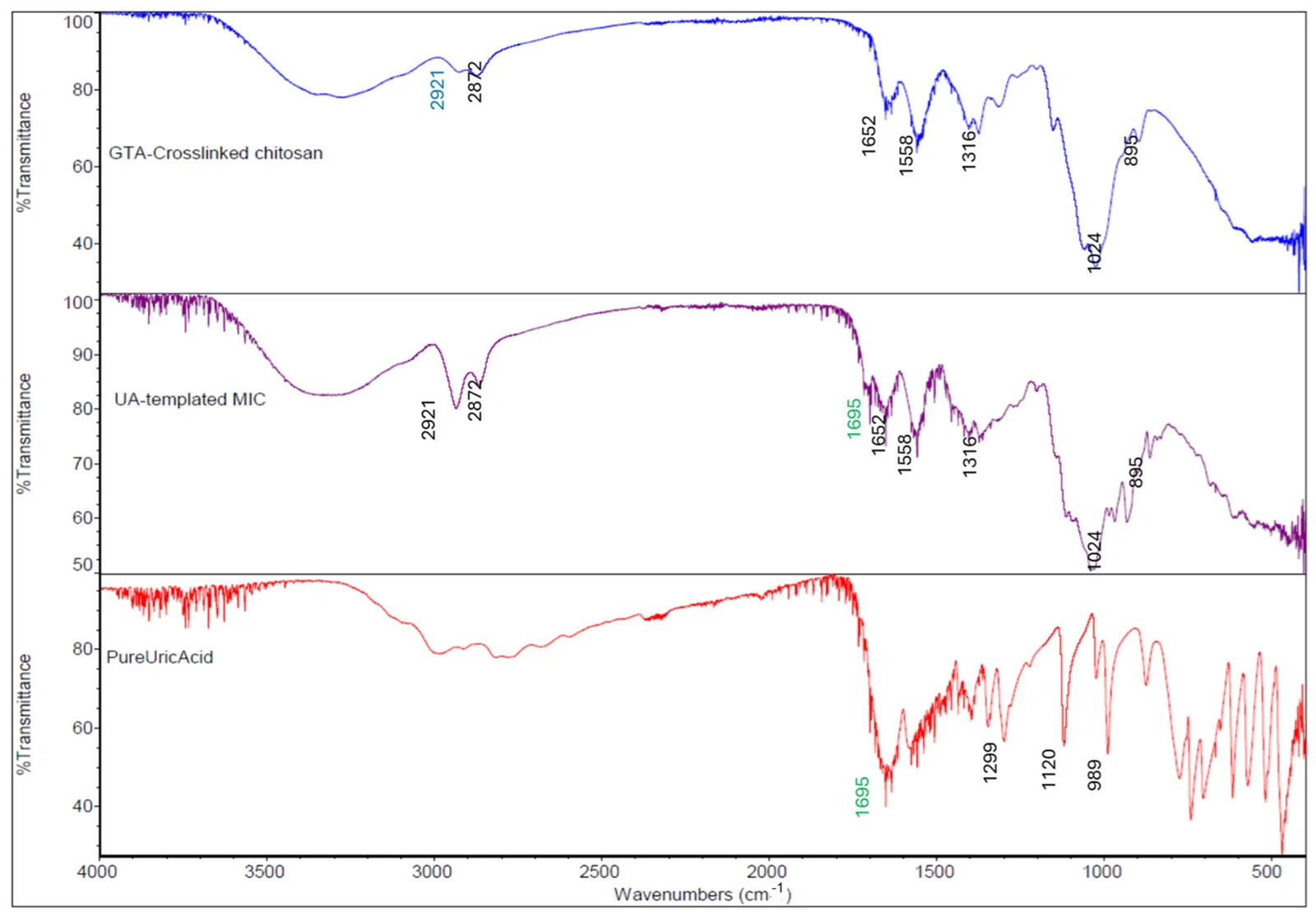
ATR-FTIR spectra of two membranes, GTA-crosslinked chitosan and UA-templated MIC, and a UA reagent sample.

FTIR spectra of three samples, a GTA-crosslinked chitosan membrane, a UA-templated MIC membrane and pure UA reagent, were recorded by loading the sample onto the iS5 instrument’s ATR adaptor and recording an FTIR transmittance spectrum for each of the samples. The recorded FTIR spectra are shown in Figure 2. Comparing the transmittance spectra of the GTA-crosslinked chitosan membrane and the UA-templated MIC membrane, a new peak at 1695 cm^−1^ was observed in the UA-templated MIC membrane’s FTIR spectrum that was absent from the GTA-crosslinked chitosan membrane’s FTIR spectrum. The peak at 1695 cm^−1^ was also observed in the pure UA reagent’s FTIR spectrum. The peak at 1695 cm^−1^ was reported to originate from C=O stretching vibration [40]. Although GTA contains C=O bonding itself, this function group in GTA reacted with the -NH_2_ group in chitosan and turned into C=N bonding in the GTA-crosslinked chitosan and UA-templated MIC membranes. Therefore, the existence of a peak at 1695 cm^−1^ in the FTIR spectra of pure UA and the UA-templated MIC membrane verified that UA was imprinted within the MIC as a template.

### 3.2. Negative Fiber Optic EW Absorption Spectra Indicating Washing out of UA from MIC Membrane

**Figure 3:**
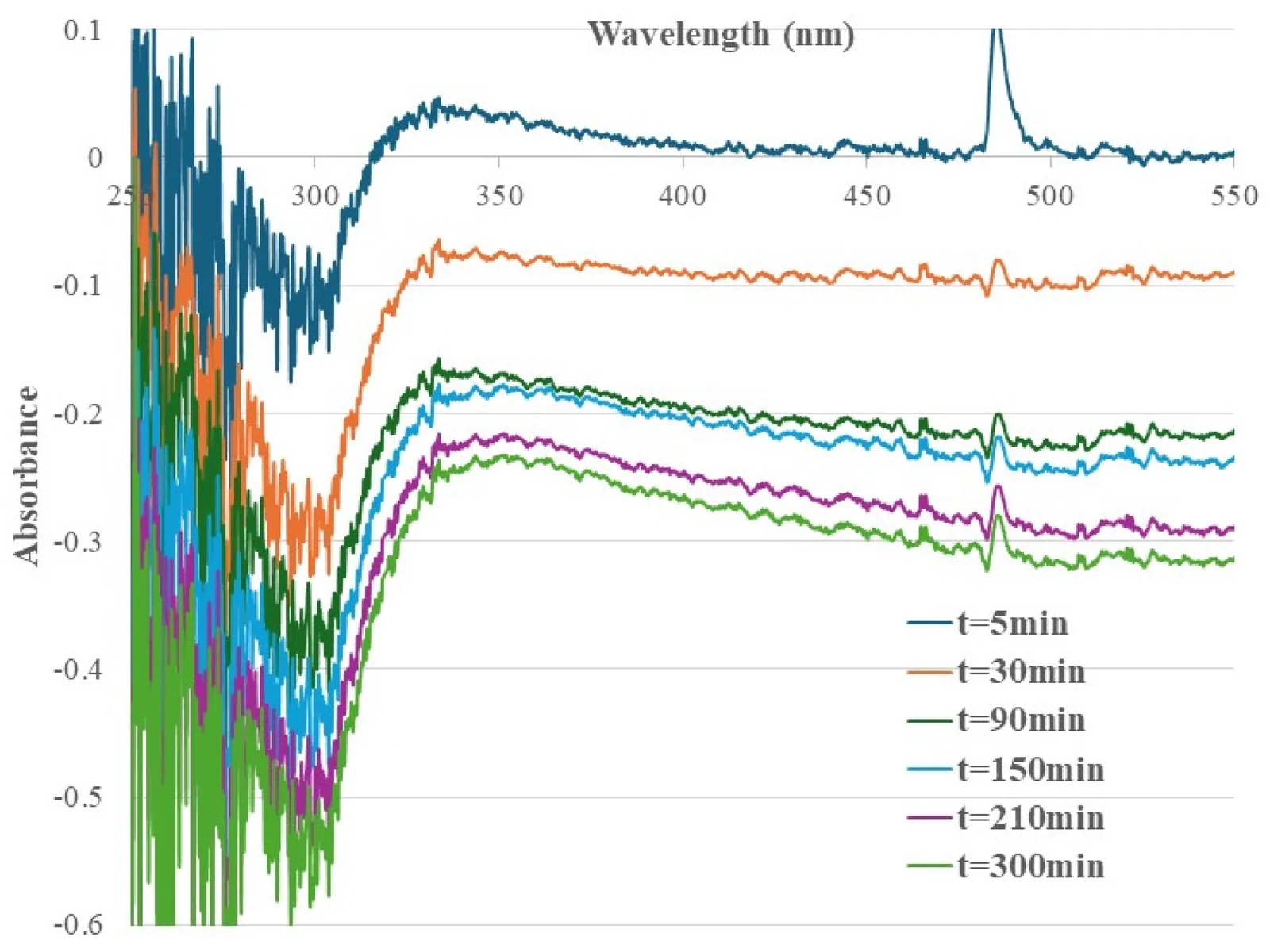
Optical fiber EW absorption spectra recorded during the time of washing out UA from the UA-templated MIC membrane by using 0.10 M NH_3_ as a washing out solution. The negative peak absorbance signal at 300 nm indicates that UA immobilized in the MIC membrane was washed out.

**Figure 4:**
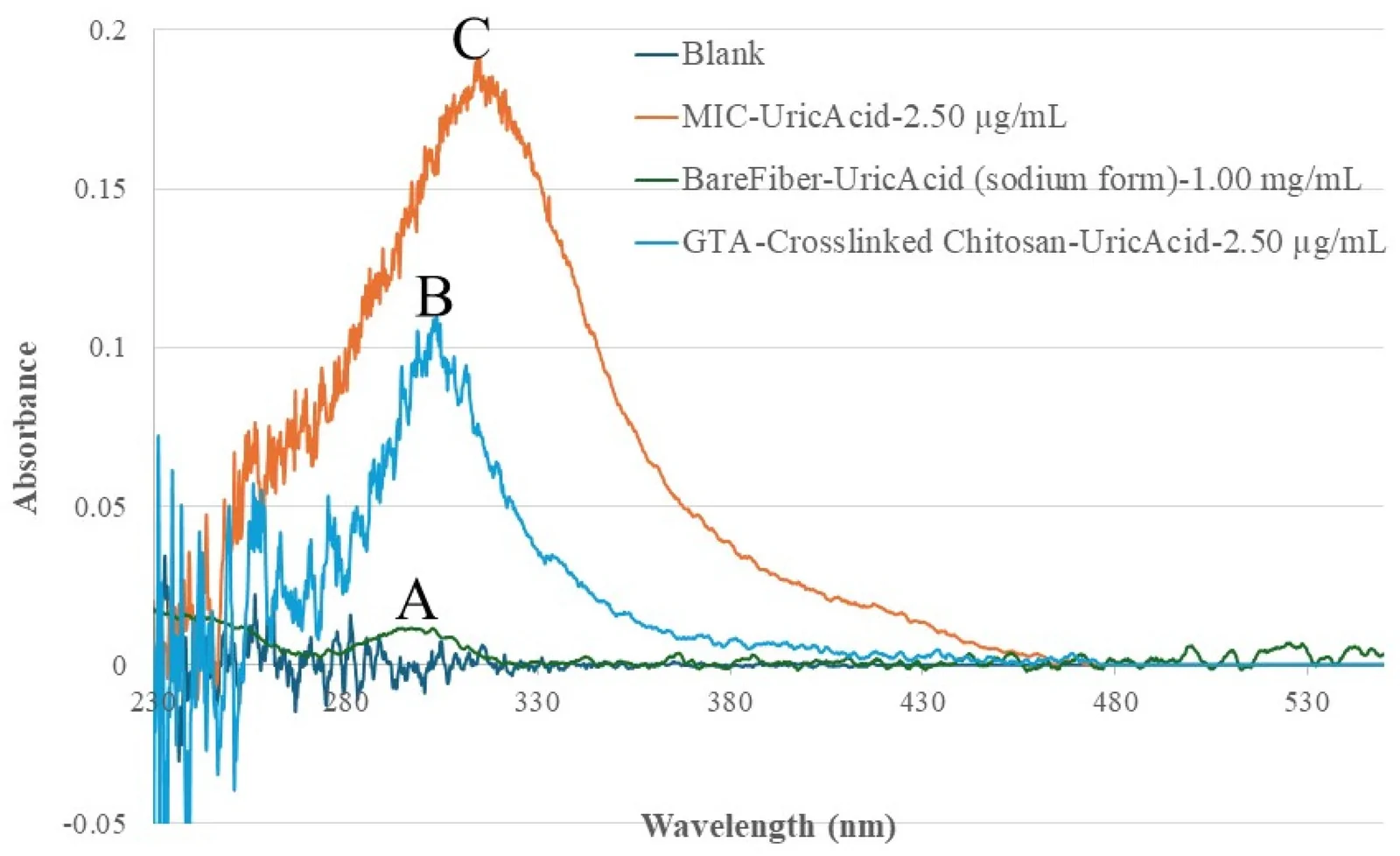
Optical fiber EW absorption spectra of a BOFP exposed to UA standard solutions. A: a bare BOFP exposed to a 1.00 mg/mL UA (sodium salt form) standard solution; B: a GTA-crosslinked chitosan-coated BOFP exposed to a 2.50 µg/mL UA standard solution; C: a UA-templated MIC-coated BOFP exposed to a 2.50 µg/mL UA solution.

The fiber optic EW absorption spectra recorded when the UA was washed out of the MIC membrane are shown in Figure 3. With the increase of time the MIC membrane-coated BOFP was inserted into the 0.10 NH_3_ solution, a negative absorption spectrum with a peak absorption wavelength at around 300 nm was observed. This negative peak absorption wavelength agrees with UA’s UV peak absorption wavelength recorded by inserting a bare fiber BOFP into a 1.00 mg/mL UA solution as shown in Figure 4 (spectrum A). Therefore, the negative absorption spectra shown in Figure 3 indicate that UA was washed out of the MIC membrane. A baseline shift in the broad wavelength range was also observed during the process of washing out UA from MIC. The baseline shift could be caused by several factors, including refractive index change due to the probe being switched from DI water to 0.10 M NH_3_ solution, the washing out of acetic acid, salt and other unreacted species from the MIC membrane. The recorded negative absorption spectra are also indirect indication of UA immobilized in MIC as a template.

UA has a very limited solubility (60 µg/mL at ambient temperature) in water. When DI water was tested for its ability to wash out UA from the MIC membrane, it was found that it takes too long a time (>24 h) to completely wash out the template. In this work, both NaOH and NH_3_ solutions were investigated for their ability to wash out UA from the MIC membrane. UA in the MIC membrane can be completely washed out within 1 h with 0.10 M NaOH as a washing out solution. However, there is a concern that NaOH causes corrosion to the silica optical fiber core, and a 0.10 M NH_3_ solution was selected as the washing out solution in this work. With 0.10 M NH_3_ as a washing out solution, UA was considered completely washed out of the MIC membrane after 2.5 h after inserting the MIC-coated probe into the washing out solution, as shown in Figure 3. The 0.10 M NH_3_ solution was also used to wash out UA after the probe was exposed to a UA-containing sample solution to re-generate the sensor probe for the next test.

### 3.3. Comparing the Sensitivity of BOFP-EW-OFCS for Analyzing UA Using a BOFP with Different Coatings

Chitosan is a hydrogel polymer. Both a GTA-crosslinked chitosan membrane (non-MIC) and a UA-templated MIC membrane can function as SPME materials to concentrate UA from an aqueous sample solution into the chitosan membranes. This SPME can significantly improve the sensitivity of fiber optic EW absorption spectrometry for analyzing UA. Figure 4 shows three BOFP EW absorption spectra. Spectrum A was recorded when a bare fiber core BOFP (without any coating) was exposed to a 1.00 mg/mL UA solution (sodium salt form). Spectrum B was recorded when a GTA-crosslinked chitosan membrane (non-MIC)-coated BOFP was exposed to a 2.50 µg/mL UA standard solution. Spectrum C was recorded when a UA-templated MIC membrane-coated BOFP was exposed to a 2.50 µg/mL UA standard solution. In all these experiments, a single BOFP was used to record the spectra. Recording all the spectra using the same BOFP ensures that the change in fiber optic EW absorption spectrometry’s sensitivity for analyzing UA in this work was caused only by the difference in the membrane (DI water, GTA-crosslinked chitosan or UA-templated MIC) on the BOFP surface [38,39]. A bare fiber BOFP was first tested. Then, a GTA-crosslinked chitosan membrane was coated on the BOFP surface and the obtained probe was tested. After that testing, the GTA-crosslinked chitosan membrane was removed from the BOFP surface by inserting the bent part of the BOFP into the K_2_CrO_4_/H_2_SO_4_ washing solution for >12 h. This re-generated BOFP was then coated with a UA-templated MIC membrane for the next test.

Comparing spectrum A and spectrum C, it was found that the SPME with the UA-templated MIC membrane increased the sensitivity of fiber optic EW absorption spectrometry for analyzing UA by 1.0 × 10^4^ times. SPME with the GTA-crosslinked chitosan membrane also increased the sensitivity of fiber optic EW absorption spectrometry for analyzing UA by 5.6 × 10^3^ times (spectra A and B in Figure 4). In the field of molecularly imprinted polymer research, a parameter called the imprinting factor (IF) was used to describe SPME improvement by molecularly imprinting the polymer against a non-molecularly imprinted polymer. The IF value obtained in this work is 1.8, which was calculated by comparing the sensitivity of fiber optic EW absorption spectrometry for analyzing UA byusing an MIC membrane-coated BOFP to that by using a GTA-crosslinked chitosan membrane-coated BOFP. Although a GTA-crosslinked chitosan membrane also extracts UA from an aqueous sample solution due to the existence of un-crosslinked -NH_2_ groups, there are more vacancies in a UA-templated MIC membrane that are specifically for extracting UA. Therefore, a UA-templated MIC membrane is more effective at extracting UA from a sample solution, improving the sensitivity of the sensor.

It was noticed that there is a shift in the peak absorption wavelength between the recorded spectra. Spectrum A’s peak wavelength is 298 nm, spectrum B’s peak absorption wavelength is 303 and spectrum C’s peak absorption wavelength is 315 nm. This peak absorption wavelength shift is caused by the change in microenvironment UA molecules exposed and UA molecule bonding with -NH_2_ in the chitosan membranes. This phenomenon was also observed in our previous works using MIC-EW-OFCS to analyze other weak organic acids [32,33,34].

### 3.4. Effect of Sample Solution Acidity on Sensor’s Response to Uric Acid

**Figure 5:**
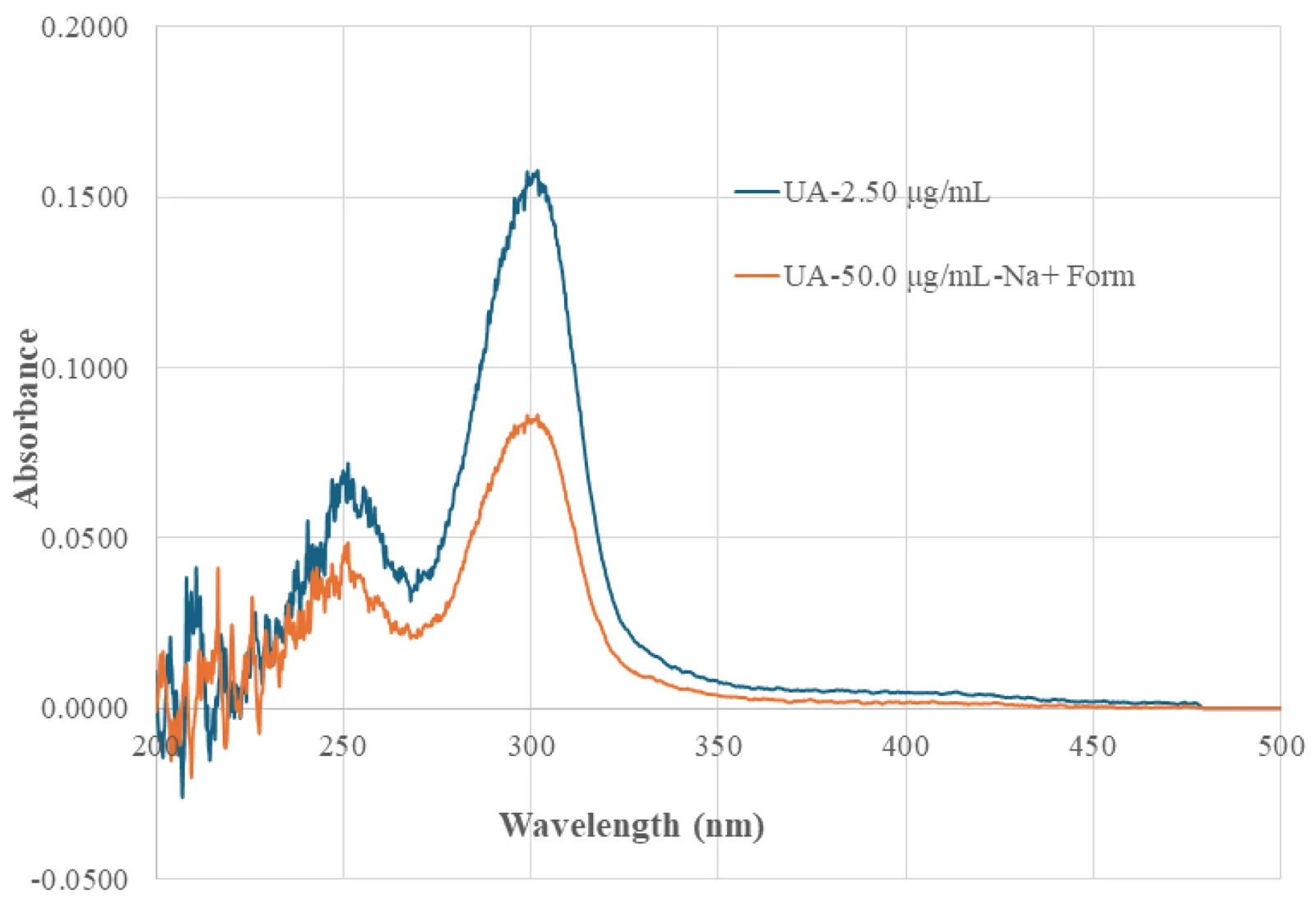
Optical absorption spectra obtained when the sensor in this work was exposed to a 2.50 µg/mL UA solution and a 50.0 µg/mL solution of UA in its Na^+^ salt form. These results demonstrate that the sensor in this work selectively responded to UA, but demonstrated much less sensitivity to the UA in its anion form.

The sensor in this work responds to UA extracted into the MIC membrane coated on the BOFP surface. The MIC membrane coated on the BOFP surface was imprinted with UA in its acid form as a template, and therefore the sensor has selective response to UA’s acid form, but is less sensitive to UA in its anion form. Figure 5 shows the recorded responses of this sensor exposed to a 2.5 µg/mL UA standard solution and a 50.0 µg/mL solution of UA in its anion form (as sodium salt). The sensitivity of this sensor to UA in its acid form is 37 times more sensitive when compared with its response to UA in its anion form. A similar phenomenon was also observed in our previous work on a molecularly imprinted chitosan-based chlorogenic acid sensor [34]. These results present some advantages of this sensor for analyzing UA in body fluid samples for medical applications. Traditional UA analysis methods, including the broadly used enzymatic methods, measure total UA (acidic form, anion form and those associated with other species) in a body fluid sample without discriminating between them. The information of free UA concentration in a body fluid sample could help in understanding UA-related stone formation in the urinate system [41,42].

**Figure 6:**
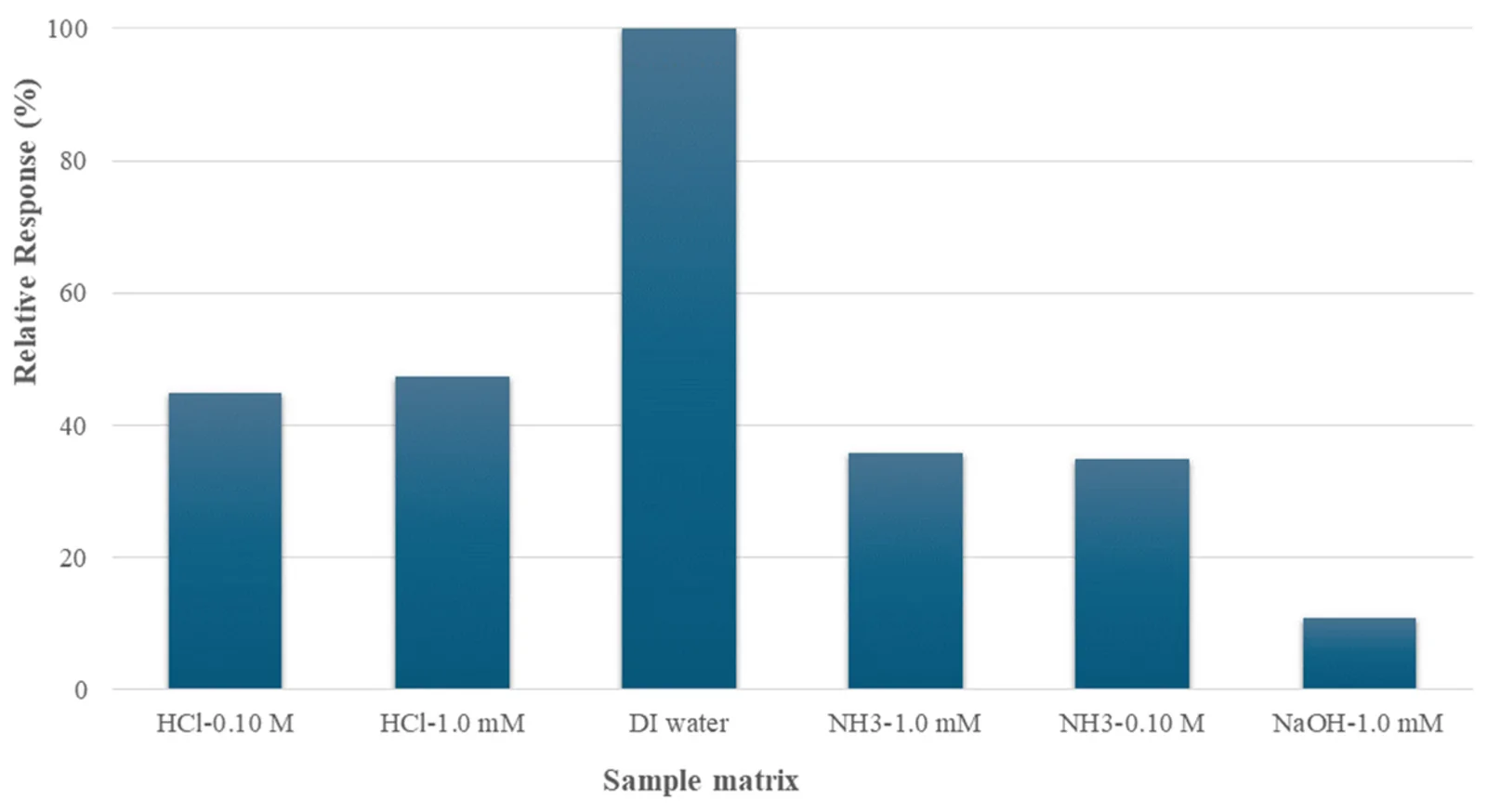
Relative response of the sensor in this work exposed to 2.50 µg/mL UA in different sample matrices. These test results demonstrate acid or base co-existing with UA shifting UA dissociation equilibrium and the equilibrium of UA bonding with amine groups in MIC.

The sensor in this work responded to UA extracted into the MIC membrane coated on the BOFP surface. Two chemical equilibria could affect this sensor’s response to UA in a sample solution: the UA acidic dissociation equilibrium and the equilibrium of the amine group (on MIC) reaction with acid. The sample solution’s acidity/basicity shifts these two equilibria. In a sample solution with high acidity, UA exists in its acidic form, which can be extracted into the MIC membrane and causes a higher response signal. However, the hydronium ions in a highly acidic sample solution also compete against UA to bond with the -NH_2_ group in the MIC, limiting the chance of extracting UA into the MIC membrane, and reducing the sensor’s response signal. On the other hand, in a higher pH value sample solution, the UA acidic dissociation equilibrium shifts toward UA’s anion forms (hydrogenurate or urate), which are not selectively concentrated into the MIC membrane. This equilibrium shift decreases the response signal of the sensor to UA in a high pH sample solution. Figure 6 shows the test results of UA (2.50 µg/mL) in different sample matrices. These test results reflect how the two equilibria affect the sensor’s response to UA as discussed above. In this work, DI water was used as sample matrix in most of the tests. The sensor is not recommended for use in analyzing UA in an acidic sample solution because the MIC membrane dissolves in acidic solutions. In a basic solution, the sensor’s sensitivity decreases because the UA’s acidic dissociation equilibrium shifts UA to its anion form, and reduces the sensor’s sensitivity.

### 3.5. Reversibility and Response Time of the MIC Membrane-Coated BOFP-EW-OFCS for Analyzing UA

**Figure 7:**
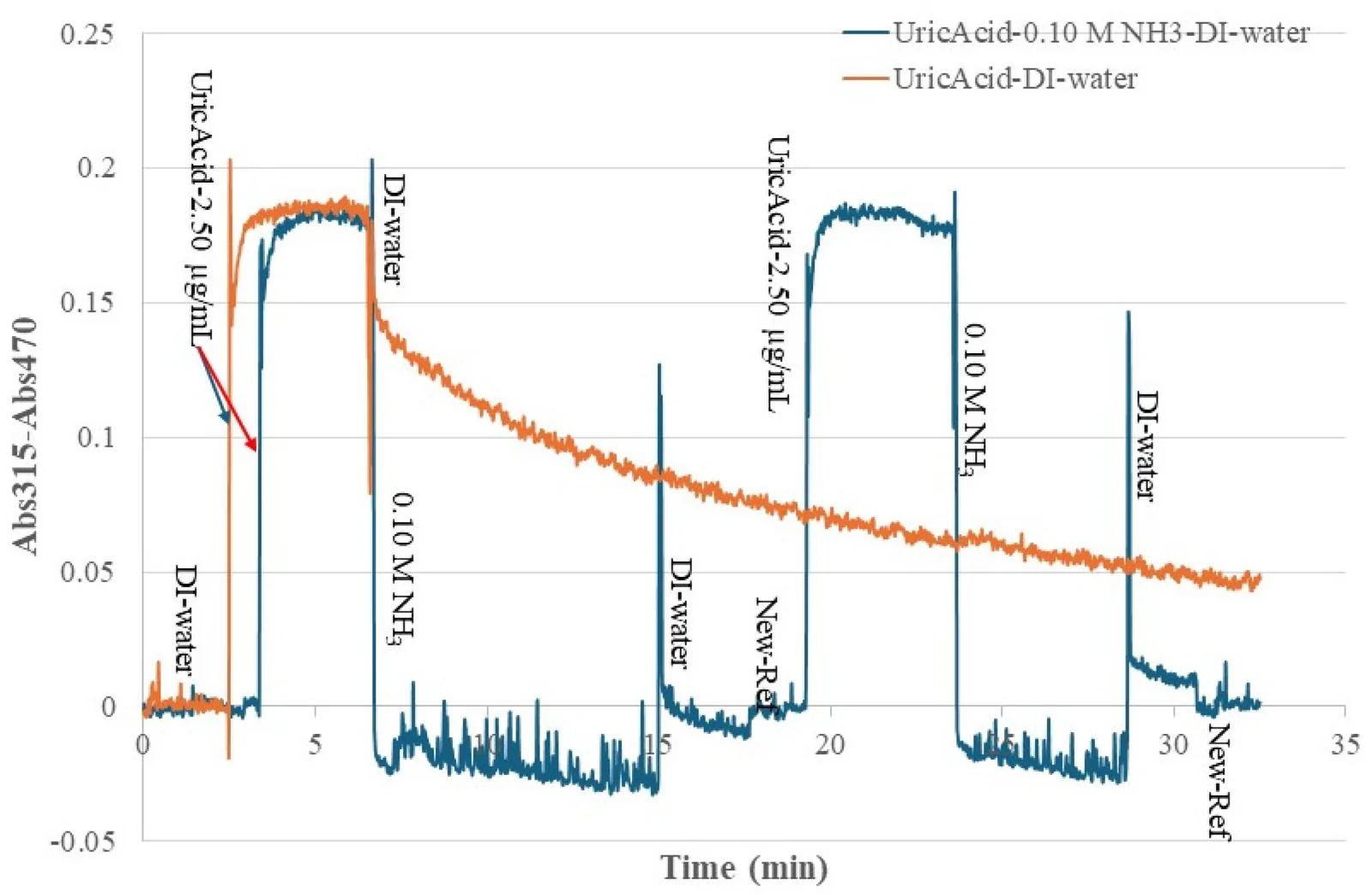
Time response of the sensor in this work alternatively exposed to DI water, a 2.50 µg/mL UA standard solution and 0.10 M NH_3_ solution.

The sensor in this work is expected to be reversible, because the sensing signal originates from SPME and the intrinsic optical absorption signal of UA itself extracted into MIC, and both are reversible processes. The reversibility and response time of the sensor was tested by alternatively exposing the sensing probe to DI water and a 2.50 µg/mL UA standard solution, and continuously monitoring the baseline-shifting corrected sensing signal (absorbance at 315 nm minus absorbance at 470 nm, Abs315-Abs470) with time. The recorded time response profiles are shown in Figure 7. The sensor demonstrates a very quick response with a response time < 1 min. However, when DI water was used to wash out UA extracted into the MIC membrane, it takes a long time to bring the signal back to the baseline due to UA’s limited solubility in water. Therefore, a 0.10 M NH_3_ solution was used to wash out UA extracted into the MIC membrane, then the sensor probe was conditioned with DI water to re-generate the sensor probe for the next test, as demonstrated in Figure 7. A sensing signal with a high noise level was recorded when the sensor probe was exposed to the 0.10 M NH_3_ solution. The reason for this noisy signal is not understood at this moment. A possible reason for the cause of this high noise is the MIC membrane’s refractive index change after it was switched to the 0.10 M NH_3_ solution, because the solution permeated to the membrane is continuously changing. However, the sensing signal noise returned to a normal level after the sensing probe was re-inserted into DI water.

### 3.6. Calibrating the MIC Membrane-Coated BOFP for Analyzing UA in Aqueous Solution Samples, and Its Detection Limit

**Figure 8:**
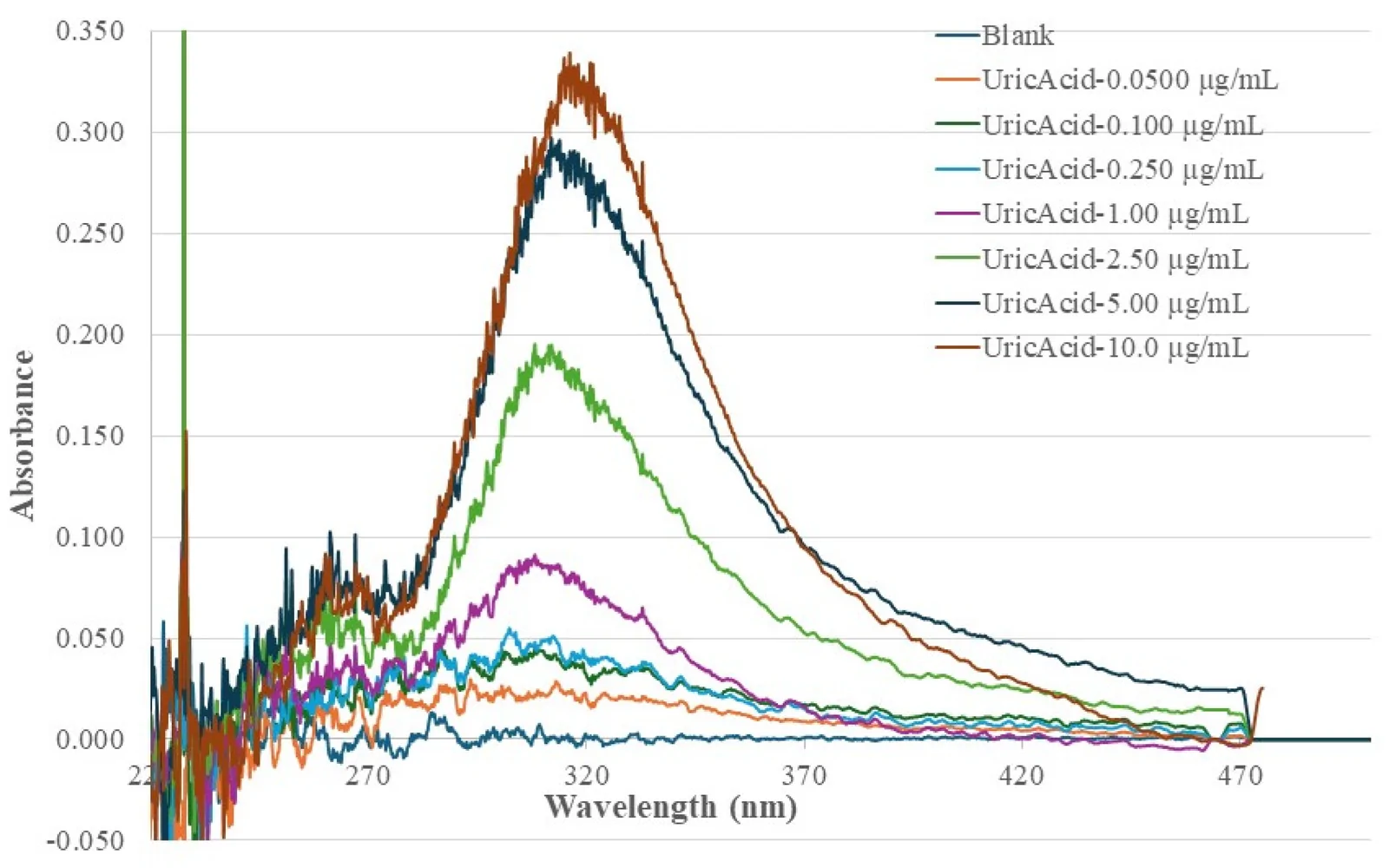
Optical fiber EW absorbance spectra of the sensor in this work exposed to UA standard solutions of different concentration.

**Figure 9:**
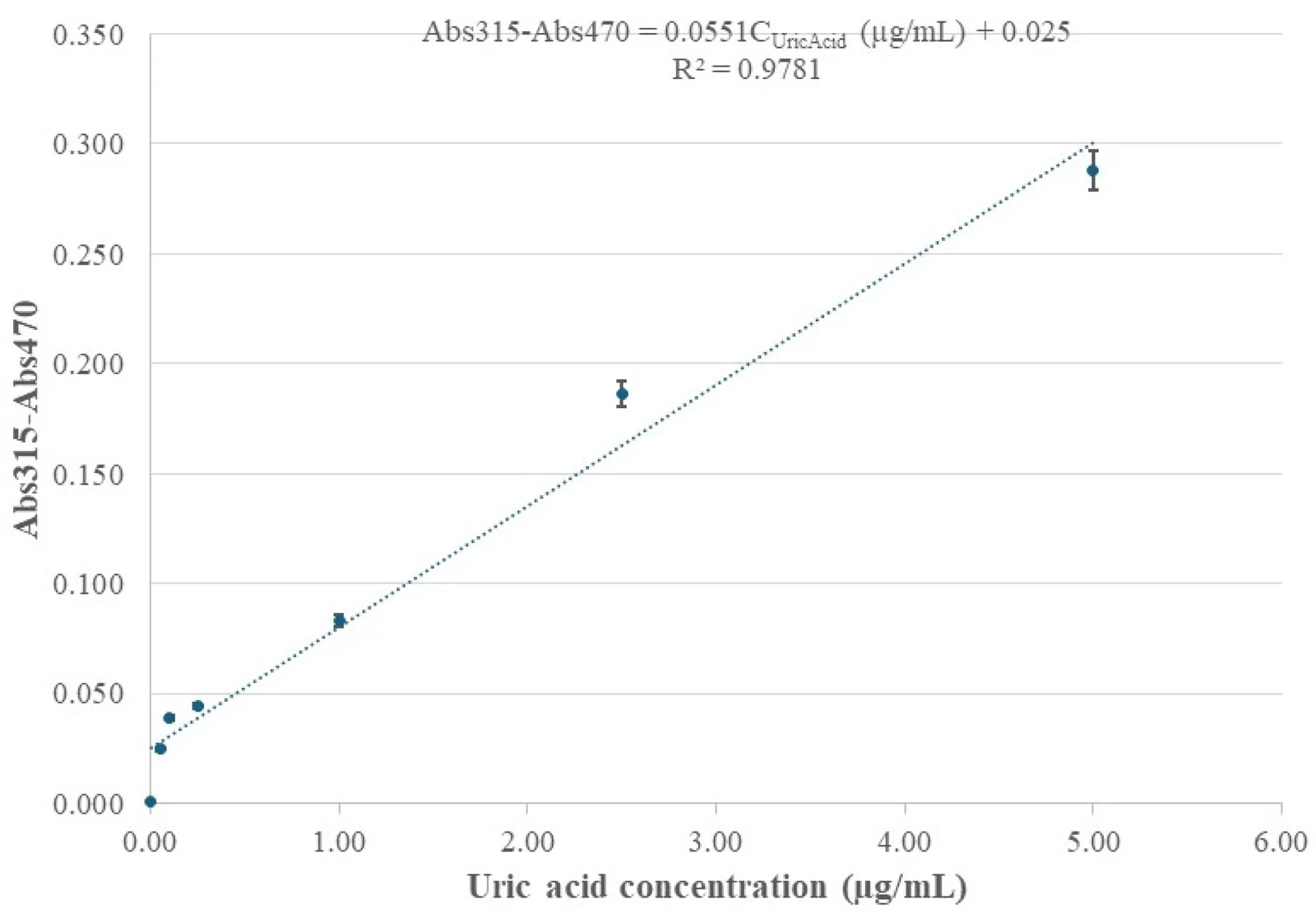
A linear calibration curve shows the relationship of the sensor’s baseline-shift corrected optical absorbance signal (Abs315-Abs470) with UA concentration in a sample solution. Each UA standard solution was tested three times and the averaged sensing signal was used in establishing this calibration curve. The error bar in each data point shows the standard deviation of the three measured sensing signals.

The relationship between the sensor’s sensing signal and UA concentration in aqueous solution samples was investigated. The BOFP coated with the UA-templated MIC membrane was exposed to UA standard solutions of different concentrations and a fiber optic EW absorption spectrum was recorded for each standard solution. Figure 8 shows the recorded spectra. With the increase of UA concentration in the standard solutions, the absorbance at 315 nm linearly increased in the tested concentration range up to 5.00 µg/mL. Above this concentration, the linear relationship between the recorded absorbance and UA concentration was not followed. The recorded spectra also show a baseline shift with increases in UA concentration. Therefore, the difference of absorbance at UA’s peak absorption wavelength (315 nm) from that at 470 nm was used as a sensing signal for quantitatively analyzing UA in sample solutions. In order to establish that a calibration curve presents the relationship between the baseline-shifting corrected sensing signal (Abs315-Abs470) and UA concentration, three sets of absorption spectra of tested standard solutions were recorded. The averaged sensing signal (Abs315-Abs470) with UA concentration up to 5.00 µg/mL was obtained by using a least-squares regression method (as shown in Figure 9).

The detection limit of this sensor is defined as UA concentration in a sample solution which causes a sensing signal that is three times the standard deviation (STDV) of a blank sample’s (DI water) sensing signal. The STDV value of the blank sample’s signal, calculated with data from the reversibility experiment (Figure 7), was 0.0009. The detection limit of this sensor was calculated using the STDV value and the slope of calibration equation to be 0.049 µg/mL. This detection limit is much lower than that of many reported methods for analyzing UA in the literature, as listed in Table 1 for comparison. The SPME concentrating UA from the sample solution significantly improved this sensor’s sensitivity and achieved a much lower detection limit. Considering the fact that UA concentration in regular people’s urine samples ranges from a few µg/mL to hundreds of µg/mL [2,3], this sensor is sensitive enough to analyze UA in regular people’s urine samples.

### 3.7. Selectivity of the MIC Membrane-Coated BOFP for Analyzing UA in Aqueous Solution Samples

The response of this sensor to some species which potentially co-exist with UA in urine samples was investigated. The following tested compounds, with tested concentrations in brackets following the compound’s name, were found not to cause the absorption signal at UA’s peak absorption wavelength (315 nm) when the MIC membrane-coated BOFP was exposed to these sample solutions: BSA (as an example of protein, 150 µg/mL), caffeine (100 µg/mL), creatinine (100 µg/mL), D-fructose (as an example of sugar, 100 µg/mL), KCl (as an example of salt, 0.010 M), NH_4_Cl (as an example of salt, 0.010 M), tryptophan (as an example of an amino acid, 100 µg/mL), urea (1.0 mg/mL). These test results demonstrate the selective nature of this sensor. An absorption spectrum was recorded when this sensor was exposed to a 100 µg/mL ascorbic acid solution, which has peak absorption wavelengths (280 nm and 340 nm) that differ from that of UA (315 nm), but will increase absorbance at UA’s peak absorption wavelength (315 nm). Although the MIC membrane was imprinted for UA, the chitosan’s amine group can react with ascorbic acid and extract ascorbic acid into the membrane to cause a sensing signal.

**Figure 10:**
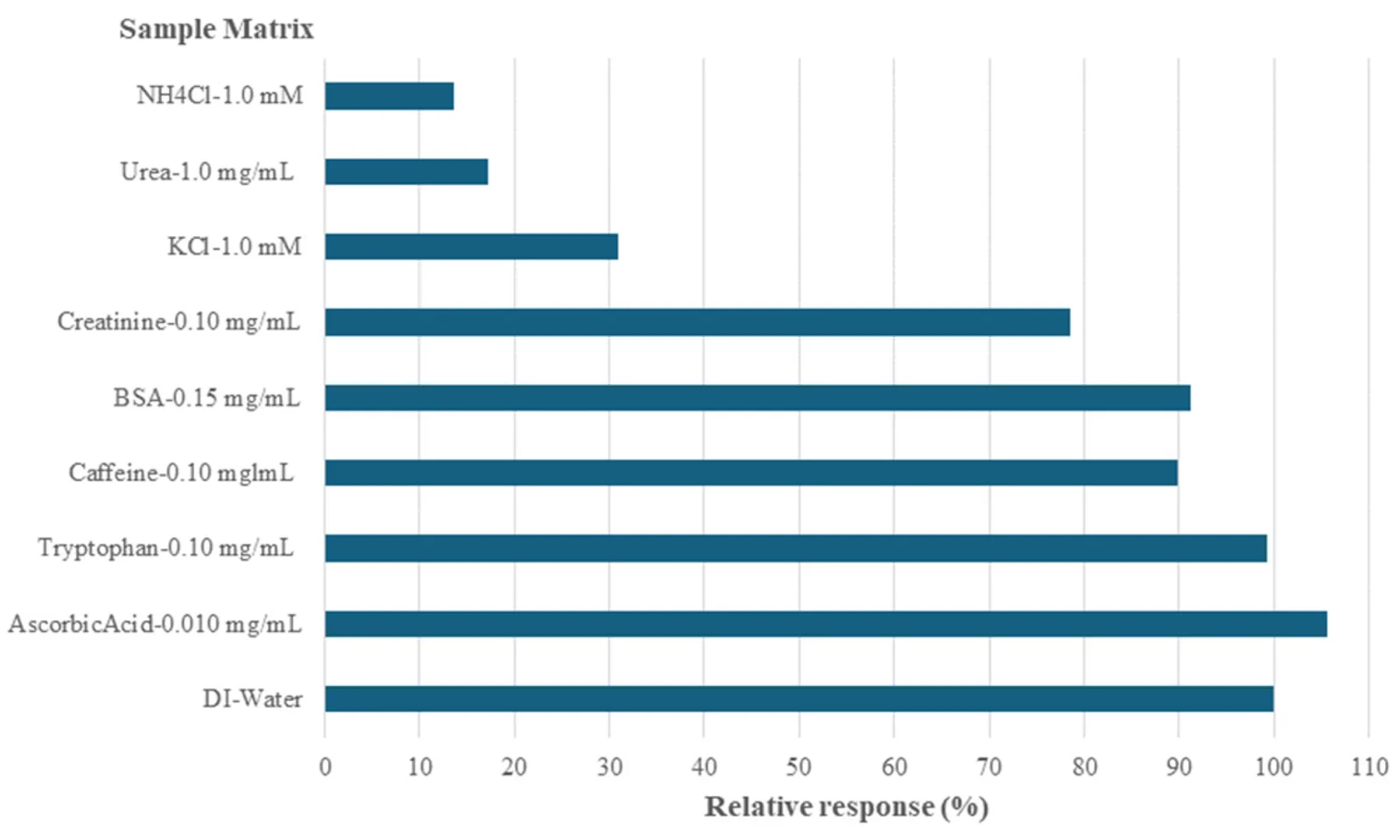
Relative response of the sensor in this work exposed to 2.50 µg/mL UA co-existing with other substances as sample matrix.

Although most of the tested substances do not cause a sensing signal at UA’s peak absorption wavelength, these substances can affect UA’s acidic dissociation equilibrium if they co-exist with UA. Figure 10 shows the relative response of 2.50 µg/mL UA added to the solutions of substances tested above. These test results indicate that the sensor’s response signal is smaller when UA co-exists with urea or creatinine. Both these compounds have amine groups which are weak bases. Therefore, UA’s acidic dissociation in these solutions shifted to hydrogenurate or urate anions. As discussed above, this sensor responds selectively to UA, but is not sensitive to UA’s anions. These test results actually illustrate the advantage of this sensor for selectively responding to UA.

The responses of this sensor to solutions of UA co-existing with KCl or NH_4_Cl are different from what was expected. Although KCl and NH_4_Cl themselves do not cause a sensing signal, the sensing signal of the sample solutions of UA co-existing with KCl or NH_4_Cl is much lower when compared with the sensor signal of UA in DI water. It was reported that ammonium hydrogenurate has very limited solubility (in the range of tens of µM) in water [41,45], which could shift UA to its ammonium salt form and result in a lower response signal. However, the effect of KCl lowering the sensor’s response to UA was not understood at this time.

### 3.8. Analyzing UA in a Urine Sample Using the Sensor in This Work

UA concentration in a urine sample donated from a volunteer donor was analyzed with the sensor used in this work. The freshly collected urine sample was immediately diluted by 40 times by adding 19.50 mL DI water to 0.500 mL urine sample. The diluted sample was immediately analyzed without any treatment and without adding any reagent. The sensor probe was inserted into the diluted test sample solution and the resulting fiber optic EW absorption spectrum was recorded. The diluted test sample was analyzed three times and the obtained results are listed in Table 2. UA concentration in the original urine sample was calculated from analytical results shown in Table 2 and the dilution factor was determined to be 88.8 µg/mL, which is within the UA concentration range of regular people’s urine samples [2,3].

Due to the limited availability of reagents and lab facilities to test a standard enzymatic method for analyzing UA in the urine sample, a standard addition/recovery test was carried out to verify the effectiveness of this sensor for analyzing UA in a urine sample. A standard addition sample was prepared by mixing 0.500 mL of the freshly collected urine sample, 2.00 mL of a 10.0 µg/mL UA standard solution, and 17.50 mL DI water. This standard addition sample was analyzed three times with the developed sensor, and the obtained results are also listed in Table 2. The obtained recovery rate ranged from 77.8% to 91.0%. The lower recovery rate was believed to have been caused by the presence of urea in the diluted urine sample solution. As discussed in Section 3.6, the existence of urea in a sample solution shifts UA’s dissociation equilibrium and part of the UA added to the sample solution was converted to UA’s anion form, which the sensor does not response to its existence. This result agrees with the fact that this sensor selectively detects UA in its acid form, but does not detect urate anions in a sample solution.

## 4. Conclusions and Further Work

The MIC-EW-OFCS developed in this work is highly sensitive and selective for analyzing UA in aqueous sample solutions, because a selective SPME-MIC membrane was integrated into the sensor’s transducer to increase UA concentration in an optical fiber’s cladding layer for fiber optic spectrometric detection. The MIC membrane also blocks out interference species from entering the optical fiber’s cladding MIC membrane, which avoids interference from species co-existing with UA in a urine sample. This sensor selectively responds to UA in its acid form, but is insensitive to UA’s anions (hydrogenurate and urate), which is different from traditional analytical methods which measure total concentration of UA and its anion form. This selectively responsive nature could provide useful information for the study of stone formation under physiological conditions. This sensor does not involve any chemical reaction for sample analysis, and no chemical reagent is needed. The analytical results of a real-world urine sample analysis verified the feasibility of this sensor to conveniently analyze UA in urine samples. In further work, we will explore the application of this sensor for analyzing UA in blood serum and other real-world samples.

## Figures and Tables

**Table 1:** Comparison between the analytical characteristics of reported UA analysis methods and the sensor used in this work.

Analytical Method	Detection Limit	Calibration Range	Samples Analyzed	Ref
UV/Vis absorption spectrometry	1.0 µg/mL	1.0–8.0 µg/mL	Urine	[16]
Electrochemistry using SiO_2_/AuNP/PANI-modified GCE	0.34 µg/mL	0.84–174 µg/mL	N/A	[43]
Bi-enzymatic colorimetry	0.5 µg/mL	0.5–12 µg/mL	Blood serum	[4]
Bi-enzymatic paper-based colorimetry	0.73 µg/mL	0.73–4.9 µg/mL	Blood serum	[44]
HPLC-UV/Vis spectrometry	0.13 µg/mL	0.25–25 µg/mL	Urine specimen	[27]
Capillary electrophoresis/chemiluminescence	0.059 µg/mL	0.10–5.0 µg/mL	Urine and serum	[29]
HPLC-TOF-MS	0.005 µg/mL	1.0–40 µg/mL	Plasma	[28]
MIC-EW-OFCS	0.049 µg/mL	0.049–5.0 µg/mL	Urine	This work

**Table 2:** Analytical results of this work’s sensor for analyzing UA in a urine sample and a standard addition urine sample.

Urine Sample (40 Times Dilution)	Obtained UA Concentration (µg/mL)	UA Added to 40 Times Diluted Sample (µg/mL)	UA Recovered (µg/mL)	Recovery Rate
Test 1	2.22			
Test 2	2.16			
Test 3	2.29			
Average	2.22			
RSD%	2.9%			
**Standard addition urine sample**				
Test 1		1.00	0.793	79.3%
Test 2		1.00	0.778	77.8%
Test 3		1.00	0.910	91.0%
Average			0.827	82.7%
RSD%			8.74%	

## Data Availability

Original data are available upon request.
